# Urinary Creatinine and Arsenic Metabolism

**DOI:** 10.1289/ehp.113-a442a

**Published:** 2005-07

**Authors:** Mary V. Gamble, Xinhua Liu

**Affiliations:** Department of Environmental Health Sciences, Department of Biostatistics, Mailman School of Public Health, Columbia University, New York, New York, E-mail: mvg7@columbia.edu

Urinary creatinine is almost universally employed to adjust concentrations of urinary analytes for variations in hydration status. In the February 2005 issue of *EHP*, Barr et al. used data from the Third National Health and Nutrition Examination Survey (NHANES III) to establish reference ranges for urinary creatinine for specific age and demographic categories (Barr et al. 2005). They reported that the significant predictors of urinary creatinine concentrations include age, sex, race/ethnicity, body mass index, and fat-free mass. Although these indicators have been known for many years, the unintentional adjustment for these covariates when urinary metabolites are expressed per gram creatinine can have profound effects on the interpretation of data. The fact that these effects are often under-appreciated or even unnoticed renders this paper highly relevant to exposure assessment and well worth revisiting. In our studies of arsenic methylation and one-carbon metabolism, we have noted several additional complications when expressing urinary arsenic as micrograms per gram creatinine. Note that one-carbon metabolism refers to the folate-dependent biochemical pathway responsible for methylation of DNA, arsenic, and hundreds of other substrates.

Our study in Bangladesh on 1,650 adults revealed that urinary creatinine concentrations are significantly correlated with plasma folate concentrations—particularly among males, who had a higher prevalence of folate deficiency than females in Bangladesh (Gamble et al. 2005). Although this association had not been previously reported, it is not surprising considering that the formation of creatine from methylation of guanidino-acetate accounts for approximately 75% of all folate-dependent transmethylation reactions (Mudd and Poole 1975) and that creatine is the direct precursor of creatinine. In some analyses, adjusting urinary arsenic for creatinine obscured correlations between folate and arsenic metabolism. In other analyses, correlations between folate and arsenic/creatinine were due in part to the associations between folate and creatinine. Correct interpretation of the data would not be possible without considering the impact of the correlation between urinary creatinine and plasma folate. As did Barr et al. (2005), we decided to include urinary creatinine in the statistical models as a separate independent variable. However, because of the intimate link between creatine metabolism and one-carbon metabolism, inclusion of urinary creatinine in some models resulted in overcontrolling for the effects of folate and homocysteine, our variables of interest. Thus, expression of total urinary arsenic per gram creatinine runs the risk of confounding relationships between total urinary arsenic and arsenic metabolism. Adjusting for the specific gravity of urine was not useful because it is so highly correlated with urinary creatinine.

In summary, we concur with Barr et al. (2005) that urinary creatinine should be included in multiple regression models as a separate independent variable; in addition, the role of one-carbon metabolism as a predictor of urinary creatinine should also be considered in interpreting results. Specifically, we routinely test if urinary creatinine itself is a predictor of the outcomes of interest.
